# (*Z*)-6-[(5-Chloro-2-hydroxy­anilino)­methyl­ene]-4-methoxy­cyclo­hexa-2,4-dienone 0.25-hydrate

**DOI:** 10.1107/S160053680901410X

**Published:** 2009-04-25

**Authors:** Arzu Özek, Orhan Büyükgüngör, Çiğdem Albayrak, Mustafa Odabaşoğlu

**Affiliations:** a Department of Physics, Ondokuz Mayıs University, TR-55139 Samsun, Turkey; b Faculty of Education, Sinop University, Turkey; c Pamukkale University, Denizli Technical Vocational School, Turkey

## Abstract

The title compound, C_14_H_12_ClNO_3_·0.25H_2_O, exists in the keto–amine form, and the aromatic rings are oriented at a dihedral angle of 7.24 (7)°. Bifurcated intra­molecular N—H⋯(O,O) hydrogen bonds result in the formation of planar six- and five-membered rings. In the crystal structure, inter­molecular O—H⋯O and C—H⋯O hydrogen bonds link the mol­ecules into chains. π–π contacts between benzene rings [centroid–centroid distance = 3.5065 (9) Å] may further stabilize the structure. There also exists a weak C—H⋯π inter­action.

## Related literature

For general background, see: Büyükgüngör *et al.* (2007 ▶); Hökelek *et al.* (2004 ▶); Odabaşoğlu *et al.* (2004 ▶). For related structures, see: Özek *et al.* (2007 ▶, 2008 ▶); Ersanlı *et al.* (2003 ▶).
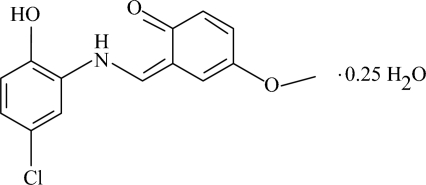


## Experimental

### 

#### Crystal data

C_14_H_12_ClNO_3_·0.25H_2_O
*M* *_r_* = 279.95
Monoclinic, 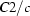

*a* = 21.3670 (11) Å
*b* = 6.7600 (3) Å
*c* = 17.7404 (9) Å
β = 103.841 (4)°
*V* = 2488.0 (2) Å^3^
*Z* = 8
Mo *K*α radiation
μ = 0.31 mm^−1^
*T* = 100 K
0.68 × 0.54 × 0.41 mm

#### Data collection

Stoe IPDS-II diffractometer
Absorption correction: integration (*X-RED32*; Stoe & Cie, 2002 ▶) *T*_min_ = 0.970, *T*_max_ = 0.970
6976 measured reflections
2588 independent reflections
2352 reflections with *I* > 2σ(*I*)
*R*_int_ = 0.022

#### Refinement

*R*[*F* ^2^ > 2σ(*F* ^2^)] = 0.031
*wR*(*F* ^2^) = 0.086
*S* = 1.09
2588 reflections
186 parameters
2 restraints
H atoms treated by a mixture of independent and constrained refinement
Δρ_max_ = 0.28 e Å^−3^
Δρ_min_ = −0.31 e Å^−3^

### 

Data collection: *X-AREA* (Stoe & Cie, 2002 ▶); cell refinement: *X-RED32* (Stoe & Cie, 2002 ▶); data reduction: *X-RED32*; program(s) used to solve structure: *SHELXS97* (Sheldrick, 2008 ▶); program(s) used to refine structure: *SHELXL97* (Sheldrick, 2008 ▶); molecular graphics: *ORTEP-3 for Windows* (Farrugia, 1997 ▶) and *PLATON* (Spek, 2009 ▶); software used to prepare material for publication: *WinGX* (Farrugia, 1999 ▶).

## Supplementary Material

Crystal structure: contains datablocks I, global. DOI: 10.1107/S160053680901410X/hk2658sup1.cif

Structure factors: contains datablocks I. DOI: 10.1107/S160053680901410X/hk2658Isup2.hkl

Additional supplementary materials:  crystallographic information; 3D view; checkCIF report

## Figures and Tables

**Table 1:** Hydrogen-bond geometry (Å, °)

*D*—H⋯*A*	*D*—H	H⋯*A*	*D*⋯*A*	*D*—H⋯*A*
N1—H1⋯O1	0.86	1.84	2.5511 (16)	140
N1—H1⋯O3	0.86	2.19	2.6063 (17)	109
C3—H3⋯O4	0.93	2.43	3.279 (5)	151
O4—H4*A*⋯O2^i^	0.831 (19)	2.029 (19)	2.842 (3)	166 (6)
O3—H3*A*⋯O1^ii^	0.852 (17)	1.743 (18)	2.5652 (16)	162 (3)
C12—H12⋯O2^iii^	0.93	2.56	3.4372 (18)	157
C7—H7*A*⋯*Cg*1^iv^	0.96	2.83	3.644 (2)	143

Symmetry codes: (i) 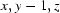; (ii) 
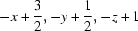
; (iii) 
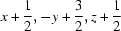
; (iv) 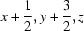. *Cg*1 is the centroid of the C1–C6 ring.

**Fig. 1.**
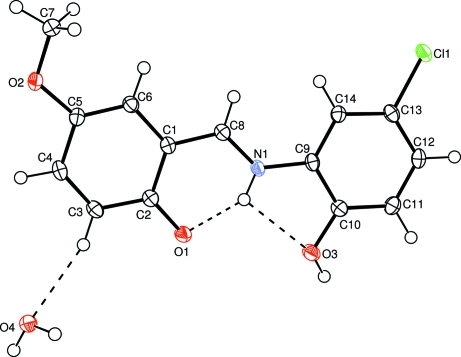
The molecular structure of the title molecule, with the atom-numbering scheme. Hydrogen bonds are shown as dashed lines.

**Fig. 2.**
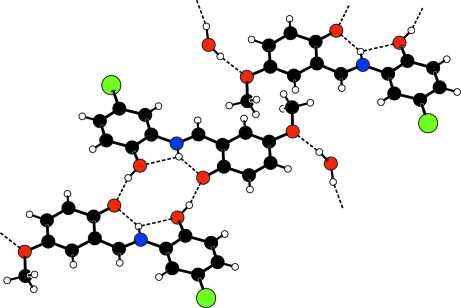
A partial packing diagram of the title compound. Hydrogen bonds are shown as dashed lines.

## supplementary crystallographic information

### Comment

As part of our ongoing studies on the syntheses and structural characterizations of Schiff-base compounds (Özek *et al.*, 2008; Özek *et al.*, 2007), we report herein the crystal structure of the title compound.

In general, *o*-hydroxy Schiff bases exhibit two possible tautomeric forms, namely, phenol-imine and keto-amine. In the solid state, the keto-amine form is observed in naphthaldimine (Odabaşoǧlu *et al.*, 2004), while the phenol-imine form is observed in salicylaldimine (Büyükgüngör *et al.*, 2007) Schiff bases. However, naphthaldimine and salicylaldimine can also exist in the phenol-imine and keto-amine forms, respectively depending on the stereochemistry of the molecule and the type of nitrogen substituents in naphthaldimine and salicylaldimine Schiff bases (Hökelek *et al.*, 2004).

In the title compound (Fig. 1), the keto-amine form is favored over the phenol-imine form, as indicated by C2—O1 [1.2924 (18) Å], C8—N1 [1.3122 (19) Å], C1—C8 [1.412 (2) Å] and C1—C2 [1.433 (2) Å] bonds. The C2—O1 and C8—N1 bonds indicate double-bond and a high degree of single-bond characters, respectively. Similar results were observed for 2-[(2-hydroxy-4-nitrophenyl) -aminomethylene]cyclohexa-3,5-dien-1(2*H*)-οne [C—O = 1.298 (2) and C—N = 1.308 (2) Å; Ersanlı *et al.*, 2003].

It is known that Schiff bases may exhibit thermochromism or photochromism, depending on the planarity or non-planarity of the molecule, respectively. Therefore, one can expect thermochromic properties in the title compound caused by planarity of the molecule; the dihedral angle between rings A (C1—C6) and B (C9—C14) is 7.24 (7)°. Intramolecular N—H···O hydrogen bonds (Table 1) result in the formations of planar six- and five-membered rings C (O1/N1/C1/C2/C8/H1) and D (O3/N1/C9/C10/H1). They are oriented with respect to the adjacent rings at dihedral angles of A/C = 4.44 (9), A/D = 9.6 (9), B/C = 5.42 (9), B/D = 2.96 (9) and C/D = 6.55 (9)°. So, they are nearly coplanar.

In the crystal structure, intermolecular O—H···O and C—H···O hydrogen bonds (Table 1) link the molecules into chains (Fig. 2), in which they may be effective in the stabilization of the structure. The π–π contact between the phenyl rings, *Cg*1—*Cg*2^i^ [symmetry code: (i) 1/2 - *x*, 3/2 - *y*, -*z*, where *Cg*1 and *Cg*2 are centroids of the rings A (C1—C6) and B (C9—C14), respectively] may further stabilize the structure, with centroid-centroid distance of 3.5065 (9) Å. There also exists a weak C—H···π interaction (Table 1).

### Experimental

For the preparation of the title compound, the mixture of 5-methoxysalicyl- aldehyde (0.5 g, 3.3 mmol) in ethanol (20 ml) and 2-hydroxy-5-chloroaniline (0.47 g, 3.3 mmol) in ethanol (20 ml) was stirred for 1 h under reflux. Crystals suitable for X-ray analysis were obtained from methanol by slow evaporation (yield; % 84, m.p. 415–416 K).

### Refinement

H atoms of water molecule and hydroxy group were located in difference Fourier maps and refined isotropically, with restrains of O3—H3A = 0.852 (17) and O4—H4A = 0.831 (19) Å. The remaining H atoms were positioned geometrically with N—H = 0.86 Å (for NH) and C—H = 0.93 and 0.96 Å, for aromatic and methyl H atoms,respectively, and constrained to ride on their parent atoms, with *U*_iso_(H) = *xU*_eq_(C,*N*), where *x* = 1.5 for methyl H and *x* = 1.2 for all other H atoms.

### Crystal data

**Table:**

C_14_H_12_ClNO_3_·0.25H_2_O	*F*(000) = 1162
*M**_r_* = 279.95	*D*_x_ = 1.495 Mg m^−^^3^
Monoclinic, *C*2/*c*	Mo *K*α radiation, λ = 0.71073 Å
Hall symbol: -C 2yc	Cell parameters from 6976 reflections
*a* = 21.3670 (11) Å	θ = 2.0–28.0°
*b* = 6.7600 (3) Å	µ = 0.31 mm^−^^1^
*c* = 17.7404 (9) Å	*T* = 100 K
β = 103.841 (4)°	Prism, red
*V* = 2488.0 (2) Å^3^	0.68 × 0.54 × 0.41 mm
*Z* = 8	

### Data collection

**Table:**

Stoe IPDS-II diffractometer	2588 independent reflections
Radiation source: fine-focus sealed tube	2352 reflections with *I* > 2σ(*I*)
plane graphite	*R*_int_ = 0.022
Detector resolution: 6.67 pixels mm^-1^	θ_max_ = 26.5°, θ_min_ = 2.0°
ω scans	*h* = −25→26
Absorption correction: integration (*X-RED32*; Stoe & Cie, 2002)	*k* = −8→7
*T*_min_ = 0.970, *T*_max_ = 0.970	*l* = −22→22
6976 measured reflections	

### Refinement

**Table:**

Refinement on *F*^2^	Primary atom site location: structure-invariant direct methods
Least-squares matrix: full	Secondary atom site location: difference Fourier map
*R*[*F*^2^ > 2σ(*F*^2^)] = 0.031	Hydrogen site location: inferred from neighbouring sites
*wR*(*F*^2^) = 0.086	H atoms treated by a mixture of independent and constrained refinement
*S* = 1.09	*w* = 1/[σ^2^(*F*_o_^2^) + (0.0376*P*)^2^ + 3.3917*P*] where *P* = (*F*_o_^2^ + 2*F*_c_^2^)/3
2588 reflections	(Δ/σ)_max_ = 0.001
186 parameters	Δρ_max_ = 0.28 e Å^−^^3^
2 restraints	Δρ_min_ = −0.31 e Å^−^^3^

### Special details

**Table:**

Experimental. 141 frames, detector distance = 100 mm
Geometry. All e.s.d.'s (except the e.s.d. in the dihedral angle between two l.s. planes) are estimated using the full covariance matrix. The cell e.s.d.'s are taken into account individually in the estimation of e.s.d.'s in distances, angles and torsion angles; correlations between e.s.d.'s in cell parameters are only used when they are defined by crystal symmetry. An approximate (isotropic) treatment of cell e.s.d.'s is used for estimating e.s.d.'s involving l.s. planes.
Refinement. Refinement of *F*^2^ against ALL reflections. The weighted *R*-factor *wR* and goodness of fit *S* are based on *F*^2^, conventional *R*-factors *R* are based on *F*, with *F* set to zero for negative *F*^2^. The threshold expression of *F*^2^ > σ(*F*^2^) is used only for calculating *R*-factors(gt) *etc*. and is not relevant to the choice of reflections for refinement. *R*-factors based on *F*^2^ are statistically about twice as large as those based on *F*, and *R*- factors based on ALL data will be even larger.

### Fractional atomic coordinates and isotropic or equivalent isotropic displacement parameters (Å^2^)

**Table:**

	*x*	*y*	*z*	*U*_iso_*/*U*_eq_	Occ. (<1)
C1	0.64391 (7)	0.8209 (2)	0.49336 (8)	0.0163 (3)	
C2	0.63352 (7)	0.6614 (2)	0.43854 (8)	0.0171 (3)	
C3	0.58193 (7)	0.6836 (2)	0.37118 (9)	0.0191 (3)	
H3	0.5736	0.5834	0.3342	0.023*	
C4	0.54480 (7)	0.8493 (2)	0.36036 (8)	0.0189 (3)	
H4	0.5112	0.8593	0.3162	0.023*	
C5	0.55578 (7)	1.0069 (2)	0.41439 (8)	0.0175 (3)	
C6	0.60491 (7)	0.9945 (2)	0.47962 (8)	0.0170 (3)	
H6	0.6129	1.0986	0.5149	0.020*	
C7	0.51514 (8)	1.3060 (2)	0.45380 (9)	0.0211 (3)	
H7A	0.5063	1.2434	0.4987	0.032*	
H7B	0.4832	1.4055	0.4349	0.032*	
H7C	0.5570	1.3663	0.4675	0.032*	
C8	0.69160 (7)	0.8057 (2)	0.56359 (8)	0.0164 (3)	
H8	0.6987	0.9125	0.5976	0.020*	
C9	0.77299 (7)	0.6029 (2)	0.65047 (8)	0.0158 (3)	
C10	0.79878 (7)	0.4110 (2)	0.65524 (9)	0.0181 (3)	
C11	0.84717 (7)	0.3589 (2)	0.71999 (9)	0.0201 (3)	
H11	0.8645	0.2321	0.7238	0.024*	
C12	0.86950 (7)	0.4950 (2)	0.77875 (8)	0.0190 (3)	
H12	0.9020	0.4604	0.8218	0.023*	
C13	0.84290 (7)	0.6831 (2)	0.77270 (8)	0.0175 (3)	
C14	0.79444 (7)	0.7395 (2)	0.70949 (8)	0.0172 (3)	
H14	0.7767	0.8657	0.7066	0.021*	
Cl1	0.873014 (18)	0.85790 (6)	0.84450 (2)	0.02270 (12)	
N1	0.72613 (6)	0.64456 (19)	0.58204 (7)	0.0165 (3)	
H1	0.7192	0.5522	0.5477	0.020*	
O1	0.66836 (5)	0.50302 (16)	0.45048 (6)	0.0219 (3)	
O2	0.51359 (5)	1.16195 (17)	0.39490 (6)	0.0229 (3)	
O3	0.77355 (5)	0.28754 (18)	0.59629 (7)	0.0236 (3)	
H3A	0.7990 (11)	0.196 (3)	0.5898 (15)	0.055 (8)*	
O4	0.5000	0.3573 (10)	0.2500	0.0253 (14)	0.25
H4A	0.500 (4)	0.286 (5)	0.2880 (13)	0.03 (2)*	0.25

### Atomic displacement parameters (Å^2^)

**Table:**

	*U*^11^	*U*^22^	*U*^33^	*U*^12^	*U*^13^	*U*^23^
C1	0.0144 (7)	0.0192 (7)	0.0150 (6)	−0.0005 (6)	0.0028 (5)	−0.0001 (6)
C2	0.0154 (7)	0.0190 (7)	0.0166 (7)	0.0000 (6)	0.0033 (5)	−0.0010 (6)
C3	0.0184 (7)	0.0226 (8)	0.0155 (7)	−0.0012 (6)	0.0023 (6)	−0.0039 (6)
C4	0.0166 (7)	0.0262 (8)	0.0127 (7)	−0.0004 (6)	0.0012 (5)	0.0000 (6)
C5	0.0158 (7)	0.0202 (7)	0.0167 (7)	0.0021 (6)	0.0043 (5)	0.0017 (6)
C6	0.0173 (7)	0.0187 (7)	0.0147 (6)	0.0001 (6)	0.0037 (5)	−0.0016 (5)
C7	0.0221 (8)	0.0180 (7)	0.0213 (7)	0.0030 (6)	0.0017 (6)	−0.0009 (6)
C8	0.0142 (7)	0.0192 (7)	0.0161 (7)	−0.0004 (6)	0.0045 (5)	−0.0018 (6)
C9	0.0119 (6)	0.0217 (7)	0.0139 (6)	0.0004 (6)	0.0031 (5)	0.0008 (6)
C10	0.0165 (7)	0.0194 (7)	0.0180 (7)	0.0003 (6)	0.0031 (6)	−0.0021 (6)
C11	0.0178 (7)	0.0196 (7)	0.0220 (7)	0.0035 (6)	0.0030 (6)	0.0025 (6)
C12	0.0153 (7)	0.0245 (8)	0.0158 (7)	0.0016 (6)	0.0011 (5)	0.0043 (6)
C13	0.0143 (7)	0.0235 (8)	0.0146 (7)	−0.0019 (6)	0.0035 (5)	−0.0017 (6)
C14	0.0158 (7)	0.0183 (7)	0.0166 (7)	0.0018 (6)	0.0021 (5)	0.0009 (6)
Cl1	0.0219 (2)	0.0258 (2)	0.01674 (19)	0.00025 (14)	−0.00266 (14)	−0.00406 (14)
N1	0.0151 (6)	0.0184 (6)	0.0142 (6)	0.0007 (5)	0.0001 (5)	−0.0018 (5)
O1	0.0205 (5)	0.0206 (6)	0.0221 (5)	0.0040 (4)	0.0002 (4)	−0.0053 (4)
O2	0.0226 (6)	0.0233 (6)	0.0189 (5)	0.0078 (4)	−0.0030 (4)	−0.0015 (4)
O3	0.0206 (6)	0.0228 (6)	0.0238 (6)	0.0049 (5)	−0.0016 (4)	−0.0077 (5)
O4	0.036 (4)	0.021 (3)	0.016 (3)	0.000	−0.001 (3)	0.000

### Geometric parameters (Å, °)

**Table:**

C1—C8	1.412 (2)	C8—H8	0.9300
C1—C6	1.426 (2)	C9—C14	1.389 (2)
C1—C2	1.433 (2)	C9—C10	1.404 (2)
C2—O1	1.2924 (18)	C9—N1	1.4050 (18)
C2—C3	1.427 (2)	C10—O3	1.3459 (18)
C3—C4	1.359 (2)	C10—C11	1.395 (2)
C3—H3	0.9300	C11—C12	1.387 (2)
C4—C5	1.415 (2)	C11—H11	0.9300
C4—H4	0.9300	C12—C13	1.386 (2)
C5—C6	1.366 (2)	C12—H12	0.9300
C5—O2	1.3720 (18)	C13—C14	1.385 (2)
C6—H6	0.9300	C13—Cl1	1.7438 (15)
C7—O2	1.4229 (19)	C14—H14	0.9300
C7—H7A	0.9600	N1—H1	0.8600
C7—H7B	0.9600	O3—H3A	0.852 (17)
C7—H7C	0.9600	O4—H4A	0.831 (19)
C8—N1	1.3122 (19)		
			
C8—C1—C6	118.69 (13)	N1—C8—H8	119.2
C8—C1—C2	120.36 (14)	C1—C8—H8	119.2
C6—C1—C2	120.91 (13)	C14—C9—C10	120.98 (13)
O1—C2—C3	121.57 (14)	C14—C9—N1	123.82 (13)
O1—C2—C1	121.51 (13)	C10—C9—N1	115.19 (13)
C3—C2—C1	116.90 (14)	O3—C10—C11	124.28 (14)
C4—C3—C2	120.85 (14)	O3—C10—C9	116.60 (13)
C4—C3—H3	119.6	C11—C10—C9	119.10 (14)
C2—C3—H3	119.6	C12—C11—C10	120.35 (14)
C3—C4—C5	121.89 (13)	C12—C11—H11	119.8
C3—C4—H4	119.1	C10—C11—H11	119.8
C5—C4—H4	119.1	C13—C12—C11	119.30 (13)
C6—C5—O2	125.86 (14)	C13—C12—H12	120.3
C6—C5—C4	119.79 (14)	C11—C12—H12	120.3
O2—C5—C4	114.34 (12)	C14—C13—C12	121.88 (14)
C5—C6—C1	119.64 (14)	C14—C13—Cl1	118.58 (12)
C5—C6—H6	120.2	C12—C13—Cl1	119.49 (11)
C1—C6—H6	120.2	C13—C14—C9	118.37 (14)
O2—C7—H7A	109.5	C13—C14—H14	120.8
O2—C7—H7B	109.5	C9—C14—H14	120.8
H7A—C7—H7B	109.5	C8—N1—C9	128.60 (13)
O2—C7—H7C	109.5	C8—N1—H1	115.7
H7A—C7—H7C	109.5	C9—N1—H1	115.7
H7B—C7—H7C	109.5	C5—O2—C7	116.07 (11)
N1—C8—C1	121.66 (14)	C10—O3—H3A	114.0 (18)
			
C8—C1—C2—O1	−2.1 (2)	C14—C9—C10—C11	−0.7 (2)
C6—C1—C2—O1	−179.69 (14)	N1—C9—C10—C11	177.86 (14)
C8—C1—C2—C3	176.39 (14)	O3—C10—C11—C12	−178.82 (15)
C6—C1—C2—C3	−1.2 (2)	C9—C10—C11—C12	−0.1 (2)
O1—C2—C3—C4	178.48 (14)	C10—C11—C12—C13	0.5 (2)
C1—C2—C3—C4	0.0 (2)	C11—C12—C13—C14	0.0 (2)
C2—C3—C4—C5	0.6 (2)	C11—C12—C13—Cl1	−177.44 (12)
C3—C4—C5—C6	0.0 (2)	C12—C13—C14—C9	−0.9 (2)
C3—C4—C5—O2	−179.47 (14)	Cl1—C13—C14—C9	176.63 (11)
O2—C5—C6—C1	178.19 (14)	C10—C9—C14—C13	1.2 (2)
C4—C5—C6—C1	−1.2 (2)	N1—C9—C14—C13	−177.27 (14)
C8—C1—C6—C5	−175.82 (14)	C1—C8—N1—C9	−176.94 (14)
C2—C1—C6—C5	1.8 (2)	C14—C9—N1—C8	−5.5 (2)
C6—C1—C8—N1	175.38 (14)	C10—C9—N1—C8	175.95 (14)
C2—C1—C8—N1	−2.3 (2)	C6—C5—O2—C7	−10.6 (2)
C14—C9—C10—O3	178.07 (14)	C4—C5—O2—C7	168.79 (13)
N1—C9—C10—O3	−3.3 (2)		

### Hydrogen-bond geometry (Å, °)

**Table:**

*D*—H···*A*	*D*—H	H···*A*	*D*···*A*	*D*—H···*A*
N1—H1···O1	0.86	1.84	2.5511 (16)	140
N1—H1···O3	0.86	2.19	2.6063 (17)	109
C3—H3···O4	0.93	2.43	3.279 (5)	151
O4—H4A···O2^i^	0.83 (2)	2.03 (2)	2.842 (3)	166 (6)
O3—H3A···O1^ii^	0.85 (2)	1.74 (2)	2.5652 (16)	162 (3)
C12—H12···O2^iii^	0.93	2.56	3.4372 (18)	157
C7—H7A···Cg1^iv^	0.96	2.83	3.644 (2)	143

Symmetry codes: (i) *x*, *y*−1, *z*; (ii) −*x*+3/2, −*y*+1/2, −*z*+1; (iii) *x*+1/2, −*y*+3/2, *z*+1/2; (iv) *x*+1/2, *y*+3/2, *z*.
